# Therapeutic Effectiveness of* Galphimia glauca* in Young People with Social Anxiety Disorder: A Pilot Study

**DOI:** 10.1155/2018/1716939

**Published:** 2018-09-27

**Authors:** Ofelia Romero-Cerecero, Ana Laura Islas-Garduño, Alejandro Zamilpa, Ma. Dolores Pérez-García, Jaime Tortoriello

**Affiliations:** Centro de Investigación Biomédica del Sur, Instituto Mexicano del Seguro Social (CIBIS-IMSS), Xochitepec, Morelos, Mexico

## Abstract

Social anxiety is one of the most common disorders found in the population attending the first level of health care.* Galphimia glauca *has been used for many years in Mexican traditional medicine to treat “nervous disorders”. A standardized extract of this species has been evaluated in clinical studies that have proven its efficacy and safety in patients with generalized anxiety disorder. In this work, a double-blind clinical trial was carried out, using sertraline as a control. Patients from both sexes (18 to 35 years old) with moderate or severe social anxiety were included. Experimental group was treated daily (orally), for 10 weeks, with an extract from* G. glauca *containing 0.374 mg/dose of Galphimine-B (G-B, active compound). Patients in the control group were given sertraline (50 mg) in the same conditions. All patients were evaluated every two weeks. Another assessment was done one month after the end of the administration period. A total of 34 patients was included, 17 in each group. Women were predominant, and the mean age was 25 ± 4.7 years. In patients who received the* G. glauca* standardized extract, a significant reduction in anxiety was observed, with a value (in the Brief Social Phobia Scale) of 41.1±10.3 points at the start and 11.2±5.6 points at the end of treatment, while patients treated with sertraline had a value of 37.7±7.3 points at the beginning and 11.1±5.2 points at the end. No significant difference was observed between the treated groups. In a similar way, the health scale showed a gradual and continuous improvement in each of the five evaluations. In conclusion, the 10-week oral administration of* G. glauca* standardized extract showed efficacy and safety in patients with social anxiety disorder, without showing a significant difference from patients treated with sertraline.

## 1. Introduction

Disability caused by mental disorders has become more important than other produced by chronic diseases due to the fact that it appears in younger people [1]. Anxiety disorders are the most frequent mental diseases present in the population. A study reported that in Latin America and the Caribbean, more than half of the patients with some mental disease had had some type of anxiety [2]. Within the classification of anxiety disorders, social anxiety is described as one of those which presents itself most frequently in the young population, but it is also placed within the category of anxiety disorders in adults with onset in childhood [3].

Epidemiological studies have shown that social anxiety is one of the most common disorders found in the general population attending the first level of health care [4–7]. Interest in social anxiety has increased in the last years, and its high prevalence has been clearly identified [8, 9]. The personality traits that are associated with social anxiety are as follows: fear of rejection, low self-esteem, feelings of inferiority, difficulty in self-affirmation, and great susceptibility to criticism and negative opinions/lack of appreciation of others [10].

The medicinal plant species* Galphimia glauca, *a native species of Mexico [11], is commonly known as “calderona amarilla” (Mexico) or* “*thryallis” (USA) and has been used in Mexican traditional medicine, for many years, to treat central nervous system disorders, specially as a nervous tranquilizer [12]. The scientific study of this plant began more than two decades ago, through different neuropharmacology models, where it became evident that the methanol extract of* G. glauca *has an inhibitory effect on the central nervous system (CNS) [13]. Through a biodirected chemical separation, a new nor-seco-triterpenoid was discovered, which was called Galphimine-B (G-B) [14]. The new compound was subjected to different pharmacological evaluations, through which it was discovered that the G-B possesses a novel action. The compound produced modifications in the neuron discharge rate of the ventral tegmental area (VTA) [15, 16]. The effects produced by G-B are selective for the dopaminergic neurons which, besides not interacting with the GABAergic system, are capable of blocking the effects produced by glutamate on the NMDA ionotropic receptors [17]. A phytopharmaceuticals, elaborated with* G. glauca *extract and standardized in its G-B content, was administered to patients who had generalized anxiety disorder. It had a substantial anxiolytic effect, very similar to that produced by lorazepam (which was used as the control treatment), but was better tolerated, since many of the patients treated with lorazepam had to withdraw from the study due to the side effects [18]. In the present work, phytopharmaceuticals prepared with a standardized* G. glauca* extract were evaluated in a double-blind clinical trial, using sertraline as a control, in young people suffering from social anxiety.

## 2. Material and Methods

### 2.1. Plant Material

The plant material used in the study, aerial parts of* Galphimia glauca* Cav., of the Malpighiaceae family, was obtained from a controlled crop in the state of Morelos, Mexico. Identification was done by M.S. Abigail Aguilar Contreras, and a voucher sample was deposited at the IMSSM Herbarium with registration number: IMSSM-11061.

### 2.2. Preparation of Plant Extract

The plant's aerial parts (10 kg) were selected and subjected to a drying procedure at room temperature and protected from light. Once dry, the material was ground with 5 HP electric equipment to obtain <5mm particles. The dry and ground material was degreased with hexane and then extracted with a 60% ethanol/water mixture at 50°C, for two hours. The solvent was eliminated from the extract totally, through a reduced pressure distillation process. The dry product was extracted in ethyl acetate and partitioned with water. The organic phase was concentrated once more and dried in high-vacuum. The final yield of the extract was 23.6%. The obtained extract was analyzed by HPLC in order to identify the G-B content. This information was needed to prepare the pharmaceutical formulation.

### 2.3. High-Performance Liquid Chromatograph Analysis (HPLC)

**Figure 1:**
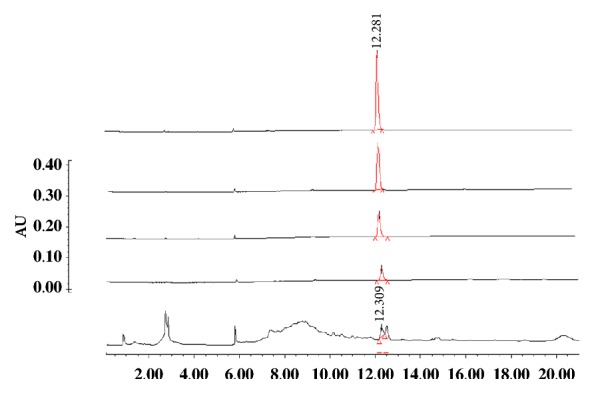
Chromatographic analysis of ascendant concentrations of galphimine-B (G-B, 25, 50 100 and 200 mg/mL) and fingerprint of anxiolytic treatment from* G. glauca*.

The dry extract of* Galphimia glauca* was analyzed in a modular HPLC system (Waters) constituted by a 2695 separation model (Alliance; Waters) and a 2996 photodiode detector (Waters). The equipment was controlled with a data capture computer software program (Empower pro; Waters). The chromatographic method was developed in a reverse-phase column (Alttima, RP- 18, 3 *μ*m, 4.6 × 70 mm; Merck). The mobile phase comprised a 35:65 acetonitrile/water isocratic system eluted at a 1.7 mL/ min flow with a 21 min run time. The fingerprints were obtained at a 220* n*m wavelength. For the calibration curve, four ascendant concentrations of G-B which were previously isolated from* Galphimia glauca* extract were injected in the same chromatographic method (Figure 3). This methodology allowed us to discover that the* G. glauca* extract contained 53 mg/g of G-B (Figure 1).

### 2.4. Preparation of Treatments

The formulation of the experimental treatment was done using the standardized* G. glauca* extract. For each dose, the amount of dry extract used was needed to reach a G-B concentration of 0.374 mg. The product was added and mixed with the vehicle in a uniform manner and then packed into hard gelatin capsules.

Sertraline was used as a control treatment, and it was purchased from a pharmaceutical supplier. In each dose, 50 mg of sertraline was used and this was added to and uniformly mixed with the vehicle. The product was packed in hard gelatin capsules that were identical to the ones in the experimental treatment.

For the secondary packaging of the capsules, experimental as well as control, 10 unit aluminum blisters were used. Both treatments were labeled to identify the research project. The containers were packed in individual cardboard boxes, also labeled with the project data and controlled through reference numbers.

### 2.5. Clinical Study

A clinical, prospective, double-blind, and randomized study was carried out, using sertraline as a control. The study population was formed by patients attending the Regional General Hospital (*Hospital General Regional*) of the Mexican Institute of Social Security (IMSS) in the state of Morelos, Mexico. Patients were assigned through a table of random numbers to one of two treatment groups. The experimental group received treatment with a standardized* G. glauca *extract and the control group was administered with sertraline.

Women and men between ages 18 and 35 were included, who had a diagnosis of moderate or severe social anxiety. The measurement instrument used for diagnosis was the Brief Social Phobia Scale (BSPS) [19], which contains 17 items, with five response options that each provides a value from 0 to 4. The sum of the values gives us the total points presented by each of the participants. When the questionnaire that was applied to the candidate showed a total value of 31 points or more (significant social anxiety), the patient was considered to be a candidate to be admitted in the study.

Once a candidate had been identified to be included in the study, a physician (who had been trained in the development of clinical projects) was in charge of performing a medical history and a medical examination in order to corroborate the diagnosis of social anxiety and the person's general state of health. Other criteria taken into account to include a candidate in the project were as follows: (a) not having had treatment for their ailment, at least for a month prior to their admittance in the project and (b) in the case of women in reproductive age, not being pregnant or breast-feeding. To formally enter the study, all patients had to sign an informed consent letter. It was also necessary to carry out clinical laboratory studies, which included the following: hematic biometry (HB), general urine test, glutamic pyruvic transaminase, glutamic oxaloacetic transaminase, urea, creatinine, and fasting blood glucose.

In the study, no patients were included who had other mental disorders, or with a history of alcoholism, smoking or drug addiction, those living alone, who took drugs for insomnia, or MAO inhibitors, patients with epilepsy, those handling dangerous machinery or who have to drive a motor vehicle for a long time.

Patients included in the experimental group were treated daily (orally), for 10 weeks, with capsules containing the* G. glauca* extract standardized in its G-B content (0.374 mg/dose). While the control group received capsules with sertraline (50 mg) during the same period of administration.

The control and experimental treatments were administered every 24 hours, during the first week and after that, twice a day, until completing ten weeks of administration. During the last two weeks, the treatments were gradually suspended.

Patients were asked to return five times (during the administration period) in order to evaluate their state of health, the evolution of the disease, and adherence to the treatment and its tolerability. During each visit, a health scale assessment instrument was applied to the patients, as well as a Clinical Global Impression scale [20]; the physician also applied this scale. In all evaluations, questions were asked about the presence of side effects, their number, and severity. At the time they joined the study and at the end of the treatment, the BSPS was applied.

At the end of the administration of the treatments, a clinical efficacy evaluation was performed, of patients treated with the phytopharmaceuticals prepared with* G. glauca *(experimental group), as well as of those who were treated with sertraline (control group). Clinical efficacy was considered to be present when the patient moved to a stage of less severity or to the total remission of symptoms. The following variables were also evaluated: (1) adherence to treatment: this variable was considered when the percentage of consumption of the treatments was equal to or greater than 75%; (2) therapeutic tolerability: this variable was considered to be the absence of severe side effects generated by the treatment, which could justify its discontinuation, (3) therapeutic success: this was considered when there was clinical efficacy and therapeutic tolerability, (4) therapeutic failure: this was considered when there was a lack of clinical efficacy and/or an absence of therapeutic tolerability.

All patients were received by the physician one month later, to evaluate the presence or absence of social anxiety symptoms.

### 2.6. Ethical Aspects

The research Project was submitted to and approved by the National Committee for Scientific Research and the National Ethics Committee of the Mexican Institute of Social Security and received registration number R-2014-785-092 from the Health Research Direction. The study was carried out according to the guidelines of the Helsinki and Tokyo Declarations for humans. Each patient included in the study received detailed information on the clinical procedure and signed a letter of informed consent.

### 2.7. Statistical Analysis

The results of the study are reported as percentages and frequencies and were analyzed through descriptive statistics, using the STATA 14 program. The X^2^ test was used for the analysis of differences in proportions and the ANOVA test for the difference in means. Values of* p *that were under 0.05 were used to define significant differences among the groups.

## 3. Results

### 3.1. Clinical Trial

A total of 34 patients were admitted to the study, of which 17 were included in each treatment group (experimental and control). Of the total number of patients in the study, 3 dropped out of the experimental group and 2 of the control group. The remaining 14 and 15 patients, respectively, continued with the treatment until the end. The statistical analysis of results was done by “intention to treat analysis” (ITT). The initial statistical analysis was applied to all patients, and this included the Brief Social Phobia Scale, and medical history. Those who continued until the end, were applied the follow-up scales: health scale assessment instrument, the Clinical Global Impression Scale and the final BSPS.

Of the total sample, 7 (20.05%) were men and 27 (79.41%) were women. The mean age was 25 ± 4.8 years. Three patients from the experimental group and two from the control group did not conclude the period of administration. In the experimental group, where patients were treated with the standardized* G. glauca *extract, two patients abandoned the study for personal reasons; among the patients treated with sertraline, there was one who dropped out, in this case due to a change in residence. Tolerability was similar in both study groups; one patient from each group had to suspend treatment due to side effects. At the end of the treatment, 82.3% (14) of patients remained in the experimental group and 88.2% (15) in the control group.

Table 1 shows the personal background and other variables related to the disease in patients included in the study. Although no significant differences are seen between the treatment groups, it is important to note that 35.2 % and 29.4% of patients in the experimental and control groups, respectively, were students, while 52.9% and 47.0%, in the same order as above, were not working. Most of the patients, 82.3% of the experimental group and 94.1% of the control group, manifested a preference for solitude.

### 3.2. Measuring Scales

**Figure 2:**
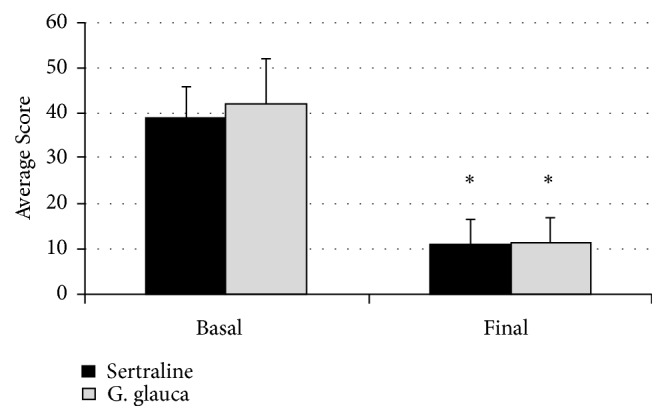
Effect produced by the oral administration for ten weeks of the standardized extract of* G. glauca *(experimental group) or sertraline (control group) on patients with a diagnosis of social anxiety, evaluated by the BSPS scale. *∗* =* p* < 0.05 when compared to the basal condition. The statistical analysis showed no difference between the treatment groups (*p* > 0.05).

**Figure 3:**
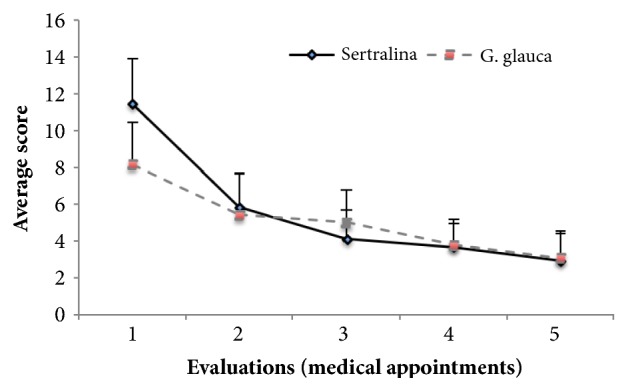
Effect produced by the oral administration for ten weeks, of the standardized extract of* G. glauca *(experimental group) or sertraline (control group) in patients with a diagnosis of social anxiety, evaluated through the health scale. The statistical analysis showed no difference between the treated groups (p > 0.05).

Using the BSPS, under basal conditions, the patients included in the study showed a maximum anxiety level of 60 points in the experimental group and 51 points in the control group. The mean per study group was 41.1 ± 10.3 points for the experimental group and 37.7 ± 7.3 points in the control group. The statistical analysis showed no evidence significant differences between the groups (*p *= 0.43). At the end of the administration period (10 weeks), the symptomatology diminished with respect to the baseline evaluation by 73.2% in the patients of the experimental group (who were treated with the standardized extract of* G. glauca*) and, 72.2% in the patients that received sertraline. As may be seen in Figure 2, we were able to determine that in patients in the experimental group, after ten weeks of treatment, anxiety was significantly reduced to 11.2 ± 5.6 points, while, in patients of the control group, it decreased to 11.1± 5.2 points. The analysis of results did not show statistically significant differences between both groups. In a similar manner, the analysis by means of the health scale shows a gradual improvement, consistent and continuous, in each one of the five appointments attended by the patients. It is important to note that the improvement in patients was observed since the second week of treatment (first medical visit after starting administration) (Figure 3).

The Clinical Global Impression Scale for improvement, answered by both the patients and the physician, showed a continuous tendency towards improvement, throughout the weeks of treatment. Results are shown in Table 2.

### 3.3. Output Variables

82.3% of the experimental group and 88.2% of the control group concluded the ten weeks of administration of treatments; of these, 14.2% (2) of patients in the experimental group and 6.6% (1) of patients in the control group achieved a total absence of symptoms related to the disease, at the end of treatment administration. The rest of the patients went on to a less severe stage, 85.7% and 93.3% in the experimental and control groups, respectively, with no evidence of a statistically significant difference between the groups (*p* = 0.54).

On average, patients in the experimental group perceived improvement in their symptoms at 15 ± 6.65 days of treatment, while the patients in the control group perceived improvement at 13 ± 7.16 days (*p* = 0.35).

Adherence to the treatment was considered to be present when the patient consumed 75% or more of the treatment. Thus, the analysis showed that adherence to the treatment among patients who concluded the study was of 100 % in both groups of treatment.

In general, the total number of patients concluding the ten weeks of treatment (29 patients) stated feeling satisfied with the response of their symptoms after treatment administration. At the end of the treatment, there was 92.8% therapeutic success in the experimental group and 93.3% (*p* = 0.54) in the control group. One patient from each group (7.1% of the experimental group and 6.6% of the control group) was identified as a therapeutic failure (*p* = 0.83).

It is important to point out that, during the withdrawal scheme (last two weeks of administration), 100% of participants who reached the end of the study, in the experimental group as well as in the control group, there was no exacerbation of the clinical profile, nor any added symptoms.

One month after conclusion of the treatment, 86.6% (13) of patients in the control group and 78.5% (11) of those in the experimental group went for a follow-up consultation. Of these, 54.5% (6) of the experimental group and 46.1% (6) of the control group did not show any symptoms that were compatible with the clinical profile of social anxiety (*p* = 0.68).

## 4. Discussion

According to the Reports of the Diagnostic and Statistical Manual of Mental Disorders-IV (DSM-IV), social anxiety is the most frequent mental disease that is found in the category of anxiety disorders in adults, with onset in childhood [3]. The results of the present study show that 76.4% (13) of patients included in the group treated with* G*.* glauca, *had had the symptoms since childhood or adolescence. The results were similar in patients treated with sertraline; in this case, the same condition was found in 88.2% (15) of participants.

Interpersonal relationships are a very important factor in people's behavior and are considered to be greatly needed for survival. These relations can be affected in some people because of the presence of social fear, which can go from inhibiting certain activities such as public speaking, facing authority figures or, in extreme cases, not initiating a new relationship [19, 20]. It is important to point out the presence of these parameters in the patients included in this study and its evolution after the treatments administration. With this purpose, a questionnaire was used to which patients could respond: absent, scarce, manageable, and unmanageable. Before starting treatment, and on the “fear that patients feel to an authority figure”, in both treatment groups the “manageable” response predominated, with 52.9% and 41.2% in the experimental and control group, respectively. After 10 weeks of treatment, the response was “absent” in 58.3% and 63.6%, in the same order. When asked about “Avoids public speaking” in both groups, before starting treatment, the most frequent response was “unmanageable” with 58.8%. At the end of the treatment, in the experimental group the response was “scarce” in 50% and absent in 35.7% of the patients; while in the control group the answer was “scarce” in 66.6% and “manageable” in 20%. Regarding the item “Fear of being with unknown people”, at the basal moment the “manageable” intensity predominated, with 52.9% in both groups. At the end of the treatment administration, the most frequent response was “scarce”, with 76.9% and 60% in the experimental and control group, respectively.

Among the patients who were administered* G*.* glauca,* the difficulty in socializing was evident, since most of them, 89.4%, said that going to parties or social gatherings was disagreeable to them; the same was stated by those who were treated with sertraline, 98.9% (*p* = 0.41).

This disease becomes specially important in adolescents due to the possible consequences that social anxiety might generate, such as: (1) low academic performance, which might lead them to drop-out of school, (2) the development of other anxiety disorders or mood problems, (3) the onset of alcohol consumption or of other toxic substances [21–23].

The population included in the study was made up by young patients, most of whom were no longer in school. Of those who were still studying, we discovered that only 50% from the experimental group, enjoyed participating in classes. In the case of the group treated with sertraline, no participants answered this question affirmatively. In the same way, 100% of the patients included in the experimental group found exams to be very stressful, a number not very different from that found in the control group (83.5%). Another figure related to this analysis was that in 100% of the participants included in both study groups, their grades did not correspond to the effort invested in their courses.

The effectiveness of sertraline has been evaluated in multiple clinical studies related to generalized anxiety or social anxiety. Time of administration has been between 10 and 24 weeks. Evaluation of evolution, using different scales has detected a response to treatment that goes from 30 to 80%. Clinical studies have found that symptoms such as fear, avoidance, restlessness, blushing, and palpitations decrease significantly with sertraline administration [24, 25].

Besides the pharmaceutical drugs used to treat anxiety problems, since ancient times, vegetal species with medicinal properties have been used as alternative treatments. Today, there has been considerable progress in the study of these species, but generally the research work involves only preclinical evaluations. For these products to be better appreciated, we need to progress in the development of medicinal prototypes through pharmaceutical formulations with scientific support, which include clinical investigations oriented towards the evaluation of their efficacy, tolerability and safety [26].

There has been continuous progress in the study of the* G. glauca* species. Basic studies in its chemistry and pharmacology have allowed for the isolation and identification of new compounds called galphimines. These compounds have also been found to possess novel mechanisms of action, specificity of action on regions of the central nervous system and on dopaminergic neurons. Also, recent studies have reported the pharmacokinetics of the* G. glauca* active compounds [27–29]. Prior clinical studies have provided evidence of anxiolytic efficacy similar to that produced by Lorazepam, one of the most widely used benzodiazepines, but with greater tolerability [30]. These findings have placed the* G. glauca *species in a relevant position within the group of species having clinical efficacy in patients with mental disorders [31]. It has been shown that galphimine-B (active compound from* G. glauca*) possesses a selective pharmacological action in regions of the CNS, such as VTA, while it also has the ability to inhibit the frequency of discharge of dopaminergic neurons. More specifically, it has the ability to block the effect produced by glutamate on dopaminergic neurons. The results obtained in this study agree with previous reports in which it has been shown that psychostimulants such as amphetamine increase the release of dopamine in VTA. This effect of dopamine, specially that generated by the discontinuation of amphetamine, affects social behavior and generates anxiety [32]. Other reports have suggested that dopamine is involved in the etiology and expression of anxiety. An assertion that is further reinforced, by the fact that nonbenzodiazepine drugs, such as dopamine antagonists, exerts clinical efficacy in patients with anxiety [33]. These data are closer to the knowledge of a probable action mechanism of G-B. It is important to highlight that the individual dose (of the active compound G-B) used in the case of the experimental treatment was considerably lower than that used for the control. Even considering the total amount of the extract included in each capsule, the dose of the experimental treatment was lower (7 mg). This means that the amount needed to produce the same effect was lower, which could mean higher pharmacological potency and, consequently, lower amount of drug administration.

In this study we were able to see that the symptoms related to anxiety, which were explored with the BSPS scale, decreased considerably after two weeks of treatment with a phytopharmaceutical prepared with a standardized extract of* G. glauca*. For example, (1) At the beginning of the study, 100% of the patients included in this group had fears, which by the end of the study decreased to 53.2%; (2) blushing was present at the initial moment in 84.2% of participants, and at the end it was present in 36.8%; (3) palpitations were present in 78.9% of patients at the beginning, and went down to 26.3% at the end; (4) Normal social activities limitations were reduced from 60 to 20%. It is important to take into consideration that in this study (avoiding living patients without medical treatment) a placebo group was not included; this situation does not allow a comparative analysis to identify a possible placebo effect.

## 5. Conclusion

The oral administration of a* Galphimia glauca *extract, standardized in its G-B content, showed efficacy and safety in patients with social anxiety disorders, without showing significant differences when compared to patients treated with sertraline.

## Figures and Tables

**Table 1:** Personal background and variables related to the disease, in patients with social anxiety who were included in the group treated with a standardized extract of *Galphimia glauca *(experimental group) and in the group treated with sertraline (control group).

Variable	*Galphimia glauca*	Sertraline	*p*
	n = 17	n = 17	X^2^
	% (frequency)	% (frequency)	
**Schooling**			
Middle School	23.5(4)	5.8 (1)	
High School	41.1(7)	57.8 (11)	0.24
University	35.2 (6)	29.4 (5)	
**Marital status**			
Single	58.8 (10)	58.8(10)	
Married	31.5 (6)	29.4 (5)	0.78
Common law marriage	5.2 (1)	5.2 (1)	
Divorced	0 (0)	5.2 (1)	
**Presently studies**			
Yes	35.2 (6)	29.4 (5)	0.71
No	68.7(11)	70.5 (12)	
**Working**			
Yes	47.0(8)	52.9 (9)	0.73
No	52.9 (9)	47.0 (8)	
**Likes to have friends**			
Yes	82.3 (14)	58.8 (10)	0.13
No	17.6 (4)	41.1 (7)	
**Feels accepted by his/her group of friends**			
Yes	70.5 (12)	76.4(13)	0.69
No	29.4(5)	23.5 (4)	
**Likes to live only with relatives**			
Yes	58.8(10)	70.5(12)	0.47
No	41.1 (7)	29.4 (5)	
**Prefers solitude**			
Yes	82.3 (14)	94.1(16)	0.62
No	17.6(3)	5.8(1)	

No significant difference was found between the two groups.

**Table 2:** Effect produced by the administration of a standardized extract of *G. glauca *(experimental group) or sertraline (control group) in patients with a diagnosis of social anxiety, evaluated at two, four, six, eight and ten weeks, using the “Patient's Global Impression Scale for Improvement” and the “Physician's Global Impression Scale for Improvement”.

	Patient's global impression scale for improvement			Physician's global impression scale for improvement		
Variable	*G. glauca*	Sertraline	*p*	*G. glauca*	Sertraline	*p*
	% (frequency)	% (frequency)		% (frequency)	% (frequency)	

**Two weeks**						
Much better	17.6 (3)	29.4 (5)		52.9 (9)	70.5(12)	0.46
Better	52.9 (9)	47.0 (8)	0.30	29.4 (5)	29.4 (5)	
Without changes	29.4 (5)	23.5 (4)		17.6 (3)	0 (0)	
**Four weeks**						
Much better	50 (7)	60 (9)		78.6 (11)	93.3 (14)	0.44
Better	21.4 (3)	33.3 (5)	0.14	7.1 (1)	6.6 (1)	
Without changes	28.5 (4)	6.6 (1)		14.2 (2)	0 (0)	
**Six weeks**						
Much better	71.4 (10)	66.6(10)		100 (14)	86.6 (13)	0.63
Better	28.5 (4)	33.3 (5)	0.62	0 (0)	13.3 (2)	
Without changes	0 (0)	0 (0)		0 (0)	0 (0)	
**Eight weeks**						
Asymptomatic	7.1 (1)	0 (0)		7.1 (1)	0 (0)	
Much better	78.5 (11)	80 (12)		92.8 (13)	86.6 (13)	0.51
Better	14.2 (2)	20 (3)	0.65	0 (0)	13.3 (2)	
**Ten weeks**						
Asymptomatic	14.2 (2)	13.3 (2)	0.18	14.2 (2)	6.6 (1)	0.18
Much better	85.7 (12)	86.6 (13)		85.7 (12)	93.3 (14)	

## Data Availability

The data used to support the findings of this study are available from the corresponding author upon request.

## Abbreviations

G-B: Galphimine-B
BEFS: Brief scale for social anxiety
CNS: Central Nervous System
VTA: Tegmental ventral area
IMSSM: Herbarium of the Mexican Institute of Social Security
HPLC: High performance liquid chromatography
HB: Hematic biometry
IMAO: Inhibitors of monoamine oxidase.
